# Gender differences in the adoption of agricultural technology: The case of improved maize varieties in southern Ethiopia

**DOI:** 10.1016/j.wsif.2019.102264

**Published:** 2019

**Authors:** Girma Gezimu Gebre, Hiroshi Isoda, Dil Bahadur Rahut, Yuichiro Amekawa, Hisako Nomura

**Affiliations:** a Department of Agricultural and Resource Economics, Graduate School of Bioresource and Bioenvironmental Sciences, Kyushu University, Japan; b Faculty of Agriculture, Kyushu University, Japan; c International Maize and Wheat Improvement Center (CIMMYT), Socioeconomics Program, El Batan, Mexico; d College of International Relations, Ritsumeikan University, Japan

**Keywords:** Gender-based decision-making, Adoption of agricultural technology, Improved maize varieties, Double-hurdle, Dawuro Zone, Ethiopia

## Abstract

This study explores the role of gender-based decision-making in the adoption of improved maize varieties. The primary data were collected in 2018 from 560 farm households in Dawuro Zone, Ethiopia, and were comparatively analyzed across gender categories of households: male decision-making, female decision-making and joint decision-making, using a double-hurdle model. The results show that the intensity of improved maize varieties adopted on plots managed by male, female, and joint decision-making households are significantly different. This effect diminishes in the model when we take other factors into account. Using the gender of the heads of households and agricultural decision-maker, the current study did not find significant evidence of gender difference in the rate and intensity of adoption of improved maize varieties. The intensity of adoption of improved maize varieties is lower for female-headed households where decisions are made jointly by men and women, compared to the male-headed households where decisions are made jointly. As the economic status is a key driver of adoption of improved maize varieties, it is recommended that the policies and programs that aim at developing and disseminating quality maize seeds in southern Ethiopia should emphatically support economically less endowed but more gender egalitarian joint decision-making households, especially female-headed ones.

## 1 Introduction

Maize (*Zea mays* L.), also called corn, is the third most important cereal crop in the world after wheat and rice (Gaya, Tegbaru, Bamire, Abdoulaye, & Kehinde, 2017), and the most widely grown crop in Ethiopia from lowland to highland agro-ecologies. Among cereals, maize accounts for the largest share in the country's crop production and is grown more than any other crop by farmers. Between 2006 and 2017, the national average yield was 2.6 tons/ha (Central Statistical Agency (CSA) of Ethiopia, 2015, Central Statistical Agency of Ethiopia, 2017), which is higher than the sub-Saharan African average of 1.98 tons/ha (FAOstat, 2018). However, if all maize farmers adopted improved maize varieties (IMVs), the maize yield could double the current level (CSA, 2017). While both male and female farmers are involved in maize production in the country, little is known about gender differences in the adoption of technologies which improve maize productivity. Low productivity of maize and other crops is still a challenge, which may be attributed to the limited adoption of improved varieties, especially by female farmers (Ragasa, Berhane, Tadesse, & Taffesse, 2012).

An extensive body of literature has examined the determinants of agricultural technology adoption (e.g., Beshir & Wagary, 2014; Doss, 2006; Doss, Mwangi, & Verkuijl, 2003; Feder, Richard, & Zilberman, 1985; Feder & Umali, 1993; Ogada, Mwabu, & Muchai, 2014; Uaiene, Arndt, & Masters, 2009; Zeng et al., 2018), however, these studies do not address gender issue in technology adoption. On the other hand, a substantial number of empirical studies have examined gender differences in agricultural technology adoption by using the sex of the household head as the gender indicator (e.g., Diiro, Ker, & Sam, 2015; Gaya et al., 2017; Kassa, Kakrippai, & Legesse, 2013). Using headship as a gender indicator is not necessarily indicative as to who actually makes decisions for agricultural production (Peterman, Behrman, & Quisumbing, 2010); hence, such studies end up dismissing the decision-making role of women within male-headed households and males within female-headed households (Doss, 2015). Women in male-headed households make production decisions often jointly with their spouses (Deere, Alvarado, & Twyman, 2009) while men (e.g., adult sons) in female-headed households also make such decisions (Bourdillon et al., 2002). Therefore, conceptualizing headship as a gender indicator does not tell us everything about the gender roles, gendered division of labour, and gender politics in agricultural technology adoption.

Some studies such as Tanellari, Kostandini, Bonabana-Wabbi, & Murray, 2014, or Addison, Aidoo, & Ohene-Yankyera, 2018, go beyond headship and look at the difference in technology adoption at the plot-manager level, using the sex of individual farmers as the gender indicator; however, this approach fails to consider gender difference in agricultural settings where plots are managed jointly by male and female farm household members and also the role of decision making in technology adoption. Thus it may lead to an incorrect policy prescription. Male and female family members face different access to inputs and information, and thus they make choices about technology adoption decisions differently. These choices may be made jointly or separately depending on the individual, household, or other conditions such as social norms and cultural dictates (Doss, 2015). Moreover, gender roles in technology adoption vary across countries and regions (Manfre, Rubin, & Nordehn, 2018). For example, in southern Ethiopia, the majority of plots are collectively farmed by households, while a few are farmed individually by males or females with little cooperation of family members.

Recently, several studies emerged that consider technology adoption decisions jointly made by men and women in the same household (e.g., Diiro, Saymour, Kassie, Muricho, & Muriithi, 2018; Lambrecht, Vanlauwe, & Maertens, 2016; Marenya, Kassie, & Tostao, 2015; Muriithi, Diiro, Kassie, & Muricho, 2018; Ndiritu, Kassie, & Shiferaw, 2014). They provide mixed evidence of gender differences in agricultural technology adoption, making it more challenging to design policies to address gender-related inequalities in agriculture. Some studies assert that there is no gender difference in technology adoption (e.g., Muriithi et al., 2018), while others claim that joint management positively influences technology adoption (e.g., Lambrecht et al., 2016; Marenya et al., 2015) or heterogeneous effects (Diiro et al., 2018). Thus, agricultural technology adoption in relation to gender is context-specific and common conclusions are challenging to reach. These studies, which focus on male and female joint management and the decision to adopt new technology are not from Ethiopia. This study seeks to provide the case of gender-based technology adoption decisions in Ethiopia with a focus on maize farming.

Using the primary data collected from four districts in Dawuro zone, southern Ethiopia, this paper provides evidence of gender-based technology adoption decisions by dividing sampled farm households into three categories: male, female and joint decision-making households for the adoption of IMVs. It asks whether there are significant differences among men, women, and joint decision-making households regarding the adoption of IMVs.^1^ It also examines factors contributing to these differences, and its implications for policies and practices in agriculture.

The rest of this paper is organized as follows: Section 2 provides a literature review, Section 3 presents methods and materials, Section 4 provides results and discussion, and Section 5 concludes the paper with policy recommendations.

## 2 Literature review

### 2.1 Gender and agriculture in Ethiopia

The most recent figures indicate that agriculture contributes to 40.5% of GDP, 81% of exports and 85% of the labour force employment among whom about half are women (Belay and Oljira, 2016). Since Ethiopia is a multicultural country, the gender roles and division of labour in agriculture varies in terms of cultural settings, locations, and farming systems (Ogato, Boon, & Subramani, 2009).

In most parts of the country, plowing with oxen and planting are primarily considered as a man's task, while women share other agricultural activities with men, such as weeding, harvesting, and collecting. The roles and decision-making power over agricultural activities of women vary across households, locations, and cultures/ethnicities in the country. For example, Belay and Oljira, 2016 note that women plow with oxen in ‘Awra Amba’ in south Gonder of northern Ethiopia while it is culturally forbidden for women to plow with oxen in central and southern Ethiopia. Gella and Tadele (2014) suggest that, even if a woman living without a husband has their own land, they cannot farm it by themselves. This holds true especially in the Dawuro community of southern Ethiopia, where female heads, even without adult men in the household, do not plow with oxen; instead, they ask non-family adult men to work on their land in the form of sharecropping.^2^ Nonetheless, because of their land ownership and involvement in sharecropping, they still retain a right to make decisions over the adoption of modern inputs on the shared cropland. Hence, gender roles in the division of labour and decision-making in agriculture maintain a level of heterogeneity in Ethiopia, due to which women and men have different extension service needs (Alemu, 2016). This variance in needs has not received sufficient attention in Ethiopian agricultural extension services (Leta, Kelboro, Stellmacher, & Hornidge, 2017).

In addition to the gender division of labour, other dimensions of gender such as social networks, asset ownership, landholding, and access to extension services also affect women's decisions over agricultural technology adoption (Seymour, Doss, Marenya, Meinzen-Dick, & Passarelli, 2016). Women in rural Ethiopia have limited access to and control over these resources (Belay and Oljira, 2016). Most notably, female-headed households operate smaller farms than their male counterparts due to a combination of resource access disadvantages; they tend to harvest lower yields than male-headed households (McMullan & Kieran, 2014).

### 2.2 Improved maize varieties in Ethiopia

There are two broad categories of IMVs: the hybrids and the open pollinated varieties (OPVs). Hybrid varieties are not supposed to be recycled because of a drop in yield potential. Contrarily, OPVs can be recycled up to a maximum of three times without significantly losing yield potential. Hybrids have a higher yield potential than OPVs (Jaleta, Yirga, Kassie, de Groote, & Shiferaw, 2013).

In 2013, >16 hybrids and 4 OPVs were under production in Ethiopia and hybrids accounted for 97% while OPVs represented only 3% of the total seed market in Ethiopia. The Ethiopian seed market has been dominated by BH660, BH540, and pioneer hybrid maize varieties (Abate et al., 2015). During the years of 2010 and 2013, the share of maize area covered by hybrids increased by 10% whiles the share of maize area covered by OPVs declined by 6% (Yirga, Bekele, & Kassie, 2017). The improved maize seeds have been diffused mainly via the Ethiopian Seed Enterprise, the major seed producer and distributor in the country (Abate et al., 2015). According to the Ethiopian Seed Association (2014), regional public seed enterprises, private seed companies, cooperative unions, and out-grower farmers have recently been getting extensively involved in the production of hybrid maize seeds. Since most of these companies are grain producers or those shifted from other businesses, they tend to have limited knowledge and skills for hybrid maize seed production, which requires special technical knowledge and management skills.

## 3 Methods and materials

### 3.1 Study area, data, and sampling

**Fig. 1:**
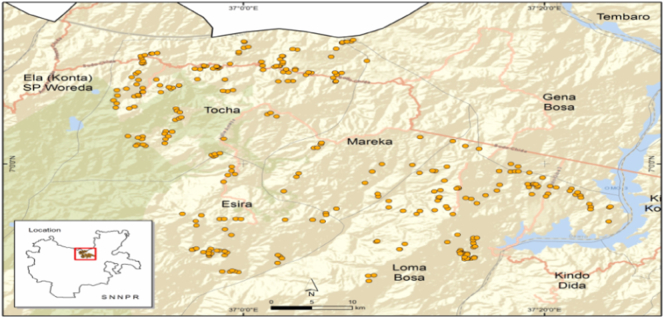
Map of the study area (Dawuro Zone) in southern Ethiopia.

Source: Authors' sketch by using GPS data (2018).

Ethiopia is divided into eleven regional administrative states, and each regional state is sub-divided into zones, woredas (districts) and kebeles.^3^ South Nations, Nationalities, and People Regional (SNNPR) state is one of the largest regional states in Ethiopia. The data used in this study was collected from Dawuro Zone in SNNPR, Ethiopia, from April to June 2018. Dawuro Zone has an altitude of 500 to 3000 m above sea level (Fig. 1). Thus, Dawuro represents a wide range of agro-ecological variations from lowland to highland. The majority of the Dawuro people live in rural areas, and their livelihood is based on a mixed crop-livestock production system. It is the major crop production area in the country and region, with maize being the major crop grown.

Multi-stage purposive sampling techniques were used to select districts, kebeles, and households in the Dawuro Zone. First, four districts, namely ‘Loma, Mareka, Esara, and Tocha’ were selected based on their maize production potentials. Second, 6–8 kebeles where maize is grown were selected from each district. Third, maize farming households in each kebele were listed and stratified into improved and local maize variety seed^4^ users; and from each stratum male and female farmers (household heads^5^) were identified. This was done with the assistance of agricultural development agents (DA) who are periodically in touch with farm households. Finally, 20 maize farm households were selected from each kebele.^6^ The number of households growing IMVs out of the selected 20 household groups varied from 6 to 14 across different kebeles. Accordingly, a total of 560 smallholder farm households (364 adopters and 196 non-adopters) were included in the survey.^7^

In all sampled households, maize farming is based on a family farming system with one or two family members^8^ (mostly wife, husband or adult sons) who make maize production decisions independently or jointly. As a cultural norm in the study area, wife and husband in the same household typically do not have separate maize farms. Hence, the person mostly responsible for farming in the household was interviewed by using a semi-structured questionnaire. Other family members were asked some supplement questions such as who makes decisions in the household about which maize variety to choose and grow.

The data collected includes information on household composition and characteristics, household membership in different social institutions, livestock ownership, off-farm incomes, inputs used for maize production and area planted, and other socio-economic characteristics of farm households in the 2017/2018 production season.^9^

### 3.2 Conceptual and methodological framework

Farm households in Ethiopia, like in any other agrarian society, need to maximize the productivity of their farmland with new agricultural inputs. Improved varieties (e.g. high yielding, disease resistant, and drought tolerant varieties) can be one of the critical agricultural inputs to maximize productivity for farm households in the developing world. The choice of the adoption or non-adoption of IMVs, along with the selection of a variety, may make a significant difference in the household trajectory and allocation of resources for maize farming in Ethiopia and elsewhere. In short, the household decision for IMV adoption may induce changes in the allocation of resources for IMVs growing and other different uses.

A simple approach to the adoption decision can illustrate this concept more clearly. This approach treats potential adopters as agents who make decisions in their own best interest.^10^ Foster and Rosenzweig (2010) note that the adoption of agricultural technology and input uses are the outcome of optimizing by heterogeneous agents. This optimization takes place in the presence of constraints on the budget, information, credit access, and the availability of both the technology, and other inputs. Hence, farm households are assumed to maximize their utility function subject to these constraints (Asfaw, Shiferaw, Simtowe, & Lipper, 2012; Foster & Rosenzweig, 2010). By following these studies, the decision around adoption is modeled in a random utility framework specified as follows:

(1) $$G_{i}^{*}={\mathrm{\beta X}}_{i}^{\prime }+\upsilon_{i}\;\mathrm{with}\;G_{i}=\left\{\begin{array}{c} 1\;\mathrm{if}\;G_{i}^{*}>0\;\\ 0\;\mathrm{otherwise} \end{array}\right)$$

where *G*_*i*_^∗^ is the latent variable representing the difference between the utility from the adopting (∪_*iA*_) and non-adopting (∪_*iN*_) of improved maize varieties, such that a utility maximizing farm household, *i*, will choose to adopt an improved maize variety if the utility gained from adopting is greater than the utility of non-adopting (*G*_*i*_^∗^ = ∪_*iA*_ − ∪_*iN*_ > 0). The term *βX*_*i*_′ provides an estimate of the difference in utility from adopting improved maize varieties (∪_*iA*_ − ∪_*iN*_); *β* is a vector of parameters to be estimated; *X*_*i*_ is a vector of explanatory variables such as individual, household and farm level characteristics; and *υ*_*i*_ is the error term.

The adoption decision process extends from hearing about the technology for the first time to actual uptake. This holds true especially of knowledge-intensive technologies such as IMVs. To understand the IMV adoption process, we assume in this study that the IMV adoption process involves two different decision stages: first, the household decides whether or not to adopt IMVs; second, the share of the maize area planted with IMVs is determined.

The probit/logit model is used to estimate whether to adopt or not while the Tobit model has been widely used to deal with the decision of how much farmland to commit to growing IMVs. However, this model is very restrictive in assuming that the two decisions are made jointly, and the same explanatory variables influence both decisions with the same sign (Greene, 2012). This assumption may not always be reasonable because the two adoption decisions could be made separately and are likely to be affected by different factors (Lin & Schmidt, 1984). Due to its joint determination, the Tobit estimators are sensitive to the violation of the underlying assumptions of homoscedasticity and normality of the errors. This could make the Tobit estimates biased and inconsistent (Greene, 2012). Because of these limitations, the Heckman (1979) selectivity model and the Cragg (1971) double-hurdle model have been suggested (Eakins, 2014; Mal, Anik, Bauer, & Schmitz, 2012; Yirga & Hassan, 2013). Both the Heckman and Cragg models relax the assumption and allow for different sets of variables to influence the two decisions. Moreover, the Cragg model further relaxes the assumption and models two decisions separately. As a result, the error terms of both decisions are jointly normal and may be correlated. In comparison to the double-hurdle model, the Heckman model is also restrictive in assuming that the zero observations arise only because of the probability of non-adoption. Meanwhile, the double-hurdle model relaxes this assumption and allows zero observations to arise in both decision processes (Eakins, 2014).

This study assumes that at the beginning of the maize production season, farm households may decide whether or not to adopt IMVs without making exact plans about the amount of maize land allocated to IMV cultivation. However, cultivation of the IMVs on each parcel of land is determined by different sets of factors. Thus, we apply the double-hurdle model instead of Heckman's. The double-hurdle model gives us more flexibility for the two sequential decisions than the Tobit and Heckman models do either respectively or collectively. The double-hurdle model postulates that two separate hurdles must be passed to gauge the proportion of the maize land planned for IMVs. The first-hurdle corresponds to factors affecting the probability of IMV adoption and the second to the intensity of IMV adoption. A different latent variable is used to model each decision process. Thus, in the first-hurdle, the probit model and in the second, the truncated regression model is applied. Following Greene (2012), Mal et al. (2012), and Eakins (2014), we specify the double-hurdle model as follows:

(2a) $$y_{i1}^{*}=z\prime_{i}\beta +\varepsilon_{i},\;\varepsilon \sim N\left[0,1\right]\;\mathrm{probability of adoption decision}$$

(2b) $$y_{i2}^{*}=x\prime_{i}\alpha +\upsilon_{i},\;\upsilon \sim N\left[0,\sigma^{2}\right]\;\mathrm{intensity of adoption}$$

(2c) $$\;y_{i}=\mathrm{\alpha x}\prime_{i}+\upsilon_{i}\;\text{if}\;y_{i1}^{*}>0\;\text{and}\;y_{i2}^{*}>0\;\text{observation mechanism}$$

(2d) $$\left(\varepsilon ,\upsilon \right)\sim \text{bvariate normal with correlation between errors}$$

where, *y*_*i*1_^∗^ is a latent variable indicating whether or not a farm household decides to adopt IMVs; *y*_*i*2_^∗^ is a latent variable describing the intensity of IMV adoption; *y*_*i*_ is the observed amount of maize area planted with IMVs; *z* and *x* are vectors of explanatory variables; *α* and *β* are independent, homoscedastic, normally distributed error terms, and *σ*^2^ is a standard deviation that incorporates heteroscedasticity across observations.

The double-hurdle model is estimated using maximum likelihood techniques with the log-likelihood function which is the sum of the log-likelihood from the probit and truncated regression (Greene, 2012). The function is specified as:

(3) $$\log\left(L\right)=\sum_{y_{i}=0}\log\left[1-Ф\left(z_{i}\beta \right)\;Ф\left(\frac{x_{i}\alpha }{\sigma }\right)\right]+\sum_{y_{i}>0}\log\left[Ф\left(z_{i}\beta \right)\sigma^{-1}\phi \left(\frac{y_{i}-x_{i}\alpha }{\sigma }\right)\right]$$

where Φ and *φ* are the standard normal cumulative distribution function and density function, respectively; *y*_*i*_ = 0 and; *y*_*i*_ > 0 represent the lower and upper corners, respectively. Since probit and truncated regressions have separate sets of parameters in the double-hurdle model, they can be estimated separately.

#### 3.2.1 Descriptions of the variables

To estimate the parameters of the model, different sets of explanatory variables are considered. The selection of these variables is based on previous empirical studies on agricultural technology adoption and available database. They are expected to influence farm household decisions on IMV adoption. The detail of those variables is given as follows.

A dummy variable represents the gender of the household head and decision makers in the household. Since male dominance in agricultural production is seen markedly in southern Ethiopia, we include these variables to examine the gender effects on IMV adoption. The use of gender to study agricultural decision-making allows us to examine the relative positions of males and females in the same household in IMV adoption.

Other household characteristics that take into account deferential impacts on IMV adoption include age and education level of the household head, and number of adult males and adult females in the household. The effect of a farmer's *age* on adoption is considered to have a composite effect of planning horizon and farming experience (Yirga & Hassan, 2013). Some studies note a shorter scope of planning with older age (e.g., Ndiritu et al., 2014); others pointed out that more experienced farmers are less likely to adopt IMVs (e.g., Mal et al., 2012). The main reason for the latter is that the longer a farmer has invested in a particular agricultural technology such as traditional seed varieties, the less likely they are to bear risks by testing a new technology such as IMVs. There is a contradictory view, however, which claims that older farmers are more likely to have access to productive resources and information, and they are more likely to adopt (Asante, Villano, Patrick, & Battese, 2017). Hence, the effect of age cannot be determined a priori.

Education could have a positive effect on the probability of IMV adoption because more educated farmers are likely to be better informed of the advantages and disadvantages of alternative technologies (Doss & Morris, 2001). Likewise, more educated farmers are more likely to have off-farm income sources while relying less on income from agriculture. They are well informed about crop diversification and balanced use of land in order to minimize risks (Mal et al., 2012).

*Household size* is used as a simple measure of labour availability, which is frequently associated with the successful adoption of new technology (Doss & Morris, 2001). However, male and female labour is not interchangeable in agriculture (Doss, 1999). For this reason, and also considering the gender division of labour in agriculture in the study area, we take into account gender differentiated labour availability by involving adult males and females as distinct explanatory variables to each other. We expect that more availability of male and female labour in the household points to a positive effect on IMV adoption.

Like many other African countries, the *ownership of livestock* is proxy for household wealth positions in Ethiopia. In the Ethiopian context, oxen and other livestock are not interchangeable for farming activities. Therefore, we take the number of oxen and other livestock units as separate explanatory variables. The ownership of oxen is an indicator of being a farmer in Ethiopia as farmers exclusively use oxen for plowing. Hence, we expect that the number of oxen owned in the household positively influences the probability and intensity of IMV adoption. On the other hand, households do not consider other livestock units as the vehicle for plowing but simply as their economic possession. Hence, the number of other livestock owned influences the probability of IMV adoption rather than its intensity. We expect that the higher the number of other livestock farmers own, the higher is the likelihood of adopting IMVs. In the area where maize is grown for livestock feed, the number of livestock may influence the intensity of IMVs. In Ethiopia, however, maize production is basically for human consumption.

Even though IMVs is scale neutral (Feder et al., 1985), the *amount of land owned* is an indicator of wealth, and it positively influences the probability of IMV adoption (Doss & Morris, 2001). On the other hand, larger farm holders tend to allocate a relatively smaller portion of their land to risky crops such as those which are more susceptible to drought. In doing so, they may earn sufficient incomes for their livelihood and hence are not willing to accept risks associated with the adoption of new agricultural technology. However, small farm holders might become the adopters of new technology if they assume that it is suited to a smaller area of land and labour-intensive work with a higher profit potential even though the technology entails some risks (Mal et al., 2012). Hence, land size owned is expected to have either a positive or negative effect on IMV adoption.

*Off-farm income and credit access* could support production activities for smallholder households. This does not always hold, however, because higher incomes from off-farm work and credit sources could change the interest of the household and they may want to divert resources away from farming activities. Hence, these variables are expected to have either positive or negative effects on IMV adoption.

*Seed sources* and *agro-ecological variation* could also influence the adoption of IMVs, and their effect would vary depending on specific extension modalities of Ethiopia. In southern Ethiopia, the IMV seed produced by different public enterprises and private companies is supplied to farmers through government extension agents without much price variation. It is up to farmers whether they use fresh IMV seeds supplied by extension agents^11^ or recycled seeds from any private sources. We assume that the seed source influences the adoption decision rather than the intensity of adoption in the study area. *Agro-ecological variation* is believed to have a profound effect on IMV adoption. In Ethiopia, different varieties of IMVs are distributed across different agro-ecologies (Abate et al., 2015). In the study area, the lowland and midland farmers allocate relatively more land to maize production than highland farmers. Similarly, lowland and midland farmers allocate a relatively larger proportion of their maize land to IMVs than highland farmers. This is because highland farmers are more experienced in growing pre-annual crops than maize production. Moreover, the highland area is more densely populated by smaller landholders than midland and lowland areas. This may explain why farmers in highland areas allocate a smaller parcel of land for maize production. This is significant because, as Slavchevska (2015) points out in Tanzania, small plot managers plant crops more densely per plot of land. Accordingly, as we move from lowland to highland agro-ecologies, the intensity of the adoption of IMVs decreases. Hence, in this study, we assume that agro-ecological variations are exogenous to the rate of IMV adoption, while endogenous to the intensity of adoption.

The variables related to extensions and social networks include contact with extension agents, market information, participation in social events, and farmer training. These variables are expected to have a positive effect on IMV adoption. The other variables, including distances from the market, extension office, and seed dealers, are expected to have negative effects on IMV adoption.

## 4 Result and discussion

### 4.1 Descriptive statistics

Table 1 provides the summary statistics of the variables of our interest. Out of all the surveyed households, 73% were headed by a male while 27% are female-headed, which is similar to the national average of 26% female-headed. In terms of gender-based decision-making, 43%, 21%, and 36% are male, female, and joint decision-making households for IMV adoption, respectively. From male-headed households, 57% and 41% are male and joint decision-making households, respectively, while the rests are female decision-making. From female-headed households, 72% and 23% are female and joint decisions making households respectively, while the rests are male decision-making. This indicates that females in male-headed households and males in female-headed households are independently or jointly making the decision on IMV adoption in southern Ethiopia. This result is consistent with studies by Bourdillon et al. (2002), Ndiritu et al. (2014) and Doss (2015). Our results imply that studies which consider only the difference in the sex of the household head would fail to capture the actual patterns of gender heterogeneity in agricultural technology adoption in Ethiopia. This said, the research shows that either a male or female, not both, is the dominant decision maker for IMV adoption in male- and female-headed households, respectively. This is consistent with studies by de la O Campos, Covarrubias, and Prieto Patron (2016) and Slavchevska (2015) in Uganda and Tanzania, respectively. Our findings might be linked to the social status of the major household decision maker as the head of household, who tends to have greater asset ownership than other family members, and who accordingly commands prominent decision-making power in the household (Deere et al., 2009).

**Table 1:** Descriptive statistics and mean differences of IMV adoption by gender of decision-maker.

Variables		Pooled [1]	Male [2]	Female [3]	Joint [4]	Test statistics		
						Difference [2] − [3]	Difference [2] − [4]	Difference [3] − [4]
*Outcome variables*								
Improved maize variety (IMV) adopter		0.65	0.68	0.63	0.65	0.05	0.03	−0.02
Proportion of maize land planted with IMVs		0.55	0.65	0.57	0.47	0.08⁎⁎⁎	0.18⁎⁎⁎	0.10⁎⁎⁎

*Independent variables*								
Female-headed household		0.27	0.05	0.72	0.23	−0.67⁎⁎⁎	−0.18⁎⁎⁎	0.49⁎⁎⁎
Male-headed household		0.73	0.57	0.02	0.41	0.55⁎⁎⁎	0.16⁎⁎⁎	−0.39⁎⁎⁎
Size of household		6.18	6.29	6.24	6.00	0.05	0.29	0.24
Number of children in the household (<15 years)		2.12	2.32	2.11	2.00	0.21	0.32	0.11
Number of males in the household (>15 years)		2.18	2.16	2.00	2.05	0.16	0.11	−0.05
Number of females in the household (>15 years)		1.88	2.06	1.89	1.95	0.17	0.11	−0.06
Age of household head in years		42.60	42.40	41.20	43.50	1.20	−1.10	−2.30⁎⁎
Education level of household head in years		3.43	3.10	3.63	3.02	−0.53	0.08	0.61
Total number of Livestock in Tropical Livestock Unit (TLU)		6.03	6.50	5.80	6.00	0.70⁎⁎	0.50⁎	−0.20
Number of oxen owned by household		1.55	1.67	1.52	1.50	0.15⁎⁎	0.17⁎⁎	0.20
Total land holding in hectares		1.58	1.73	1.55	1.42	0.18⁎⁎	0.31⁎⁎⁎	0.13⁎⁎
Size of land planted with maize in hectares		0.82	0.96	0.90	0.78	0.06⁎⁎	0.18⁎⁎⁎	0.12⁎⁎⁎
Access to credit service (1 = yes)		0.38	0.36	0.35	0.33	0.01	0.03	0.02
Contact with extension agent (1 = yes)		0.78	0.80	0.76	0.78	0.04	0.02	−0.02
Participation in social activities (1 = yes)		0.66	0.70	0.63	0.68	0.07	0.02	−0.05
Participation in farmer training (1 = yes)		0.72	0.72	0.70	0.75	0.02	0.03	−0.05
Distance from market in km		10.90	10.63	11.34	10.90	−0.71⁎⁎	−0.27⁎⁎	0.44⁎⁎
Distance from maize seed dealer in km		2.10	2.20	2.30	1.90	−0.10	0.30⁎⁎	0.44⁎⁎
Distance from Agriculture extension office in km		2.11	2.18	1.95	2.12	0.23	0.06	−0.17
Access to market information (1 = yes)		0.71	0.70	0.67	0.75	0.03	−0.05	−0.08
Access to off-farm income (1 = yes)		0.08	0.05	0.07	0.12	−0.02⁎	−0.07⁎⁎	−0.05⁎⁎⁎
Maize seed source (1 = government)		0.65	0.64	0.67	0.66	−0.03	−0.02	0.01
Type of improved maize varieties used	BH540	0.77	0.48	0.50	0.52	0.125	0.323	−0.02
	BH660	0.10	0.08	0.07	0.03	0.01⁎⁎	−0.05⁎⁎⁎	0.04⁎⁎⁎
	Pioneer	0.13	0.06	0.08	0.09	−0.02⁎⁎⁎	−0.03⁎⁎⁎	−0.01
Agro-ecology	Lowland	0.57	0.56	0.50	0.60	0.06	−0.04	−0.10
	Midland	0.38	0.38	0.39	0.38	−0.01	0.00	0.01
	Highland	0.05	0.05	0.10	0.02	−0.05⁎⁎⁎	0.03⁎⁎	0.08⁎⁎⁎
Number of observations		560	240	118	202			

⁎⁎⁎ Level of significance at 1%.

⁎⁎ Level of significance at 5%.

⁎ Level of significance at 10%.

On average, about 65% of sampled households grow IMVs with 55% of their total maize area planted with IMVs. These results are in line with the findings by Muricho et al. (2013), who engaged in the *Pathways to Sustainable Intensification in Eastern and Southern Africa* project. They found that the rate and the intensity of IMV adoption in Ethiopia are 65.40 and 58.58% respectively. However, our results are lower than the findings reported by Yirga et al. (2017) involved in the *Sustainable Intensification of Maize and Legumes Cropping Systems for Eastern and Southern Africa (SIMLESA)* project. They found that the average rate and the intensity of IMV adoption in selected areas of Ethiopia are 86.08 and 71% respectively.

With respect to the gender of the decision maker, without indicating any causal relationship, Table 1 shows that there are no significant gender differences in the adoption status of IMVs. This result is consistent with what Ndiritu et al. (2014) found in Kenya. However, there is a statically significant gender difference in the proportion of maize area planted with IMVs. The proportion of maize area planted with IMVs is significantly higher in the plots managed by male decision-making households, and lower in the plots managed by joint decision-making households. These results are opposite to what Marenya et al. (2015) and Muriithi et al. (2018) found in Uganda and Kenya, respectively. A possible explanation for our result is that the distributed hybrid maize seeds (e.g., BH540, BH660, and pioneer) in the study areas are highly susceptible to climate-related risks such as drought, disease, and insect pests.

The Ethiopian Seed Association (2014) points out that maize farmers are well aware of the benefits of using a hybrid maize seed. However, a lack of quality seed is one of the critical constraints to increasing production and productivity. In our study area, non-adopter farm households reported that because of the lack of the ability of IMVs to resist the harsh environmental conditions of a dry spell during which insect pests and disease spread, they do not prefer using these improved varieties of maize seeds. Instead, they use a local maize variety which they believe is highly adaptable and resistant to insect pests in both the field and store. According to some of them, the local variety is a low yielder, but they have adopted it based on the development of previously used or recycled hybrid varieties. Farmers are not content with the drought-intolerant existing improved maize seeds and are demanding one which is highly resistant to the harsh environmental conditions and pest attacks. The prospect of continuous growth of maize production and productivity through technological innovation is uncertain in Ethiopia under climate variability (Abate et al., 2015). Another possible explanation would be that the joint decisions by male and female family members are more strategically effective than a unilateral decision to choose a less risky agricultural technology in Ethiopia.

The average size of the family in the sampled household is 6.18, which is higher than the national average of 4.80, which may be due to regional variation. The average numbers of adult male and female family members are higher in male decision-making households, whereas they are fewer in female decision-making households, without significant differences. The average age of head in joint decision-making households is significantly higher than female decision-making households. The average number of years of household head's education for the sampled households is 3.43. However, it is higher in female decision-making households than male and joint decision-making households.

The average size of landholding for sampled households is 1.58 ha, which is higher than the national and regional average holdings of 1.02 ha and 1.23 ha, respectively. The average land owned^12^ by male and female decision-making households is 1.73 ha and 1.55 ha, respectively, indicating 0.18 ha difference with statistical significance. However, the difference is higher with joint decision-making households, which is 1.42 ha or 0.31 ha less than male decision-making households. This implies that joint decision-making households own smaller sized land than male and female decision-making households. In Ethiopia, smallholder land is often located in proximity to their home garden (Abebe & Mulu, 2017). Women tend to be the major cultivators and caretakers of the home garden, and they would be more likely to cooperate with men to work the land located near the home garden. Hence, those women might have more say in making decisions about agricultural technology adaption than the case of larger landholders whose land cultivation is more dominated by men.

**Fig. 2:**
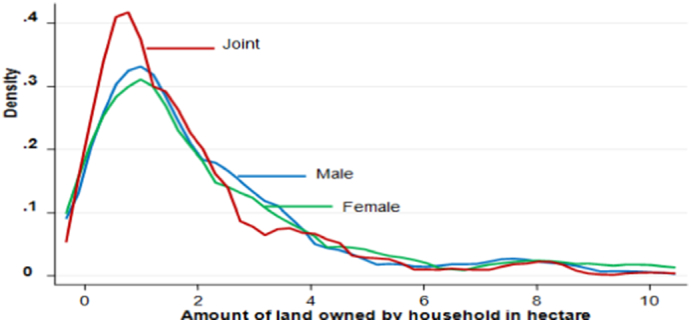
Landholding distributions of male, female, and joint decision-making households.

Source: Authors computation from the survey result (2018).

The gender difference in landholding becomes ever more apparent when we compare the landholding distribution among male, female, and joint decision-making households (Fig. 2). The distribution of landholding for joint decision-making households is predominantly at the left of that for male and female decision-making households. However, in the lower middle and right-tail of the distribution, the distributions for male, female, and joint decision-making households nearly overlap. The average land devoted to maize cultivation in the 2017/18 production season by the male, female, and joint decision-making households is 0.96 ha, 0.90 ha, and 0.78 ha, respectively. This implies that the maize plots managed by male decision-making households are larger in size, which is consistent with the findings of Muriithi et al. (2018) in Kenya.

The average number of livestock (measured in Tropical Livestock Unit)^13^ owned by male decision-making households is significantly higher than female decision-making households. Likewise, the number of oxen owned by male decision-making households is significantly higher than female decision-making households. These indicate that male decision-making households have higher asset holdings than female decision-making households. The superior ownership of oxen by male decision-making households implies that, in the study area, men are more likely to use animal traction than female decision-making households are.

The variables related to agricultural extension, social capital, and networks such as access to credit service, contact with extension agents, participation in social events, participation in farmer training, access to market information, and distance from agricultural extension offices, are not significantly different among male, female and joint decision-making households. However, there is a statically significant gender difference in access to off-farm income and distances from the market. Joint decision-making households have more access to off-farm income compared to male and female decision-making households. This result could be related to the smaller landholding status of joint decision-making households, which would suggest that these households engage more in off-farm income earning activities due to the limited land they own and cultivate for sustaining their livelihoods. The main types of off-farm income earning activities reported by sampled households are small trading, remittance, and handicrafts.

### 4.2 Estimation results

The empirical results obtained from estimating the double-hurdle model is presented in Table 2, Table 3. In both hurdles, we estimated pooled and separate samples. For a pooled sample, we estimated two models. In Model 1, female household heads and in Model 2, female decision-maker are included as gender variables respectively. We also include the interaction between female head and joint decision-making in pooled Model 2, to predict gender heterogeneity in IMV adoption. In Model 1, the female head dummy lacks a significant explanatory power while in Model 2, the interaction between joint decision-making and female-headed negatively and significantly influence the intensity of IMV adoption (Table 3). To examine the relative positions of males and females in the same household, we estimated male, female, and joint decision-making households for IMV adoption. In the first hurdle separate estimation, the sex of female-headed lacks a significant explanatory power while in the second hurdle, the sex of female-headed is negatively associated with joint decision-making for the intensity of IMV adoption. The negative and significant effect of female-headed and joint decision-making suggests that the decision around the intensity of IMV adoption made jointly by men and women in female-headed households is less than that of male-headed households.

**Table 2:** Double-hurdle models of factors influencing the IMV adoption decision for full sample and gender of the decision makers.

Hurdle 1: Probability of IMV adoption: Probit regression	Pooled model 1	Pooled model 2	Male	Female	Joint
Female head	−0.091 (0.154)		0.123 (0.773)		−0.322 (0.307)
Female decision maker		−0.013 (0.184)			
Female head ∗ joint decision maker		−0.244 (0.306)			
Children	0.018 (0.046)	0.006 (0.045)	0.034 (0.077)	0.036 (0.100)	−0.036 (0.081)
Adult male	0.124⁎⁎ (0.057)	0.119⁎⁎ (0.057)	0.015⁎⁎ (0.089)	0.143 (0.131)	0.202⁎⁎ (0.098)
Adult female	−0.135⁎⁎ (0.065)	−0.141⁎⁎ (0.065)	−0.020 (0.110)	−0.358⁎⁎ (0.173)	−0.196⁎ (0.118)
Age of head	−0.003 (0.007)	−0.003 (0.007)	0.011 (0.014)	0.020 (0.020)	−0.022⁎⁎ (0.011)
Education of head in years	0.025 (0.025)	0.029 (0.025)	0.020 (0.042)	0.095⁎⁎ (0.054)	0.023 (0.042)
Total land holding in ha.	0.125⁎⁎⁎ (0.044)	0.122⁎⁎⁎ (0.044)	0.158⁎ (0.083)	0.059 (0.070)	0.089 (0.071)
Land planted with maize in ha.	0.013 (0.041)	0.009 (0.041)	0.012 (0.081)	0.097 (0.351)	0.046 (0.212)
Seed source (1 = government)	0.089 (0.125)	0.075 (0.128)	0.145 (0.205)	0.113 (0.230)	0.096 (0.216)
Credit	0.580⁎⁎ (0.232)	0.588⁎⁎⁎ (0.230)	0.851⁎⁎⁎ (0.362)	0.335 (0.397)	0.451 (0.445)
Participation in social events	0.055 (0.147)	0.071 (0.146)	−0.124 (0.232)	0.325 (0.364)	0.107 (0.240)
Distance from main market	−0.031⁎⁎ (0.013)	−0.030⁎⁎ (0.013)	−0.052⁎⁎ (0.023)	−0.019⁎ (0.023)	−0.029 (0.021)
Distance from seed dealer	−0.068 (0.043)	−0.068 (0.044)	−0.142⁎ (0.074)	−0.020 (0.081)	−0.075 (0.081)
Distance from extension office	−0.007 (0.036)	−0.006 (0.036)	−0.055 (0.054)	−0.014 (0.097)	0.032 (0.063)
Market information	0.876⁎⁎⁎ (0.153)	0.858⁎⁎⁎ (0.151)	0.905⁎⁎⁎ (0.249)	0.817⁎⁎⁎ (0.366)	0.496⁎ (0.258)
Off-farm income	−0.515⁎⁎ (0.252)	−0.534⁎⁎ (0.253)	−0.495 (0.518)	−0.277 (0.738)	−0.763⁎⁎ (0.330)
Extension contact	0.845⁎⁎⁎ (0.214)	0.831⁎⁎⁎ (0.216)	0.950⁎⁎⁎ (0.360)	0.632 (0.561)	1.078⁎⁎⁎ (0.369)
Participation in farmer training	0.908⁎⁎⁎ (0.199)	0.916⁎⁎⁎ (0.198)	0.988⁎⁎⁎ (0.326)	0.865⁎⁎ (0.465)	0.780⁎⁎ (0.324)
Oxen	0.032 (0.101)	0.038 (0.101)	0.043 (0.193)	−0.005 (0.244)	0.062 (0.176)
Other livestock in TLU	0.032⁎ (0.020)	0.032⁎ (0.019)	0.048 (0.033)	0.033 (0.042)	0.035 (0.031)
Constant	−2.564⁎⁎⁎ (0.465)	−2.495⁎⁎⁎ (0.457)	−4.194 (0.861)	−2.279 (1.383)	−1.556⁎⁎⁎ (0.694)
Wald chi2	185.82	192.73	82.90	82.90	151.16
Prob > chi2	0.000	0.000	0.000	0.000	0.000
*N*	560	560	240	118	202

Standard errors are given in parentheses.

⁎⁎⁎ Level of significance at 1%.

⁎⁎ Level of significance at 5%.

⁎ Level of significance at 10%.

**Table 3:** Hurdle 2: Intensity of IMV adoption: Truncated regression.

Proportion of maize land planted with IMVs	Pooled model 1	Pooled model 2	Male	Female	Joint
Female head	0.034 (0.136)		−0.027 (0.351)		−0.281⁎ (0.153)
Female decision maker		0.125 (0.131)			
Female head ∗ joint decision maker		−0.432⁎⁎ (0.177)			
Children	0.026 (0.040)	0.031 (0.043)	0.011 (0.064)	0.039 (0.086)	0.071 (0.056)
Adult male	0.170⁎⁎⁎ (0.047)	0.173⁎⁎⁎ (0.050)	0.146⁎⁎ (0.079)	0.119⁎⁎ (0.085)	0.238⁎⁎⁎ (0.069)
Adult female	0.006 (0.060)	0.012 (0.060)	−0.012 (0.083)	−0.105 (0.126)	0.014 (0.079)
Age of head	−0.010⁎ (0.007)	−0.010⁎ (0.006)	−0.003 (0.011)	−0.006 (0.016)	−0.017⁎⁎⁎ (0.006)
Education of head in years	−0.038⁎ (0.021)	−0.039⁎⁎ (0.020)	0.005 (0.031)	−0.037 (0.027)	−0.055⁎ (0.033)
Credit	−0.574⁎⁎⁎ (0.191)	−0.549⁎⁎⁎ (0.151)	−0.775⁎⁎⁎ (0.234)	−0.202 (0.218)	−0.500⁎⁎ (0.229)
Land holding in hectares	0.024⁎ (0.016)	0.020 (0.025)	0.016 (0.014)	0.012 (0.024)	−0.033⁎⁎ (0.013)
Participation in social events	−0.381⁎⁎⁎ (0.126)	−0.375⁎⁎⁎ (0.125)	−0.428⁎ (0.209)	−0.123 (0.231)	−0.322⁎⁎ (0.163)
Market information	0.976⁎⁎⁎ (0.237)	0.965⁎⁎⁎ (0.233)	0.833⁎ (0.389)	0.925⁎⁎⁎ (0.321)	0.939⁎⁎⁎ (0.287)
Off-farm income	−0.372 (0.226)	−0.381 (0.253)	−0.100 (0.465)	−0.537 (0.361)	−0.215 (0.282)
Extension contact	0.094 (0.289)	0.111 (0.263)	0.176 (0.377)	0.493 (0.597)	0.292 (0.266)
Participation in farmer training	0.196 (0.220)	0.168 (0.228)	0.780⁎⁎ (0.361)	0.210 (0.407)	0.012 (0.242)
Distance from main market	−0.048⁎⁎⁎ (0.015)	−0.045⁎⁎⁎ (0.014)	−0.077⁎⁎⁎ (0.023)	−0.045⁎⁎ (0.023)	−0.007⁎⁎ (0.016)
Distance from maize seed dealer	−0.006 (0.031)	−0.001 (0.028)	−0.004 (0.047)	−0.009 (0.052)	−0.021 (0.042)
Distance from extension office	0.005 (0.036)	0.001 (0.032)	−0.038 (0.044)	−0.031 (0.098)	0.031 (0.035)
Oxen	0.487⁎⁎⁎ (0.050)	0.489⁎⁎⁎ (0.050)	0.500⁎⁎⁎ (0.092)	0.529⁎⁎⁎ (0.065)	0.372⁎⁎⁎ (0.082)
Lowland agro-ecology	0.009 (0.212)	0.050 (0.260)	0.396 (0.478)	−0.198 (0.333)	−0.038 (0.316)
Midland agro-ecology	−0.132 (0.208)	−0.088 (0.254)	−0.115 (0.469)	0.075 (0.309)	−0.070 (0.303)
Constant	−0.261 (0.584)	−0.387 (0.637)	0.052 (0.954)	−0.969 (1.191)	−0.066 (0.712)
/Sigma	0.751⁎⁎⁎ (0.051)	0.749⁎⁎⁎ (0.062)	0.795⁎⁎⁎ (0.096)	0.632⁎⁎⁎ (0.102)	0.556⁎⁎⁎ (0.067)
N	364	364	157	76	131

Standard errors are given in parentheses.

⁎⁎⁎ Level of significance at 1%.

⁎⁎ Level of significance at 5%.

⁎ Level of significance at 10%.

#### 4.2.1 Factors affecting adoption decision

Table 2 presents the pooled and separate estimation results of the probit model that determines the factors influencing the probability of IMV adoption. In pooled models, the number of adult males and adult females in the household is positively and negatively associated with the probability of IMV adoption, respectively. This suggests that the availability of male labour in the household could positively influence IMV adoption while female labour could negatively influence IMV adoption. This might be related to the conventions of the agricultural gender division of labour in Ethiopia. In the study area, maize cultivation is operated using ox plowing, which requires physical strength from male muscle force. For this reason, land preparation is considered as an exclusively male task while food preparation, child care, and other home activities are perceived as female tasks.

The land size is positively associated with the probability of IMV adoption. This is consistent with the findings of Doss and Morris (2001). This suggests that it may not be economically efficient for smallholder households with small land sizes to adopt IMVs because the amount of improved seeds contained in a pack^14^ is too much for their economic needs. Another explanation is that, since land is an indicator of wealth, an increase in land size increases the likelihood of adopting IMVs. Other factors such as access to credit, access to market information, and participation in farmer training and number of livestock owned are also positively associated with the probability of IMV adoption. This is consistent with the previous studies of Ragasa et al. (2012) and Asfaw et al. (2012). However, distances from the market and off-farm income are negatively associated with the probability of IMV adoption. These results are consistent with what Yirga et al. (2017) found. A possible explanation for this negative relationship between off-farm income and IMV adoption is that, as households gain more access to off-farm income, they tend to shift their focus and effort from maize investment to investing in other agricultural sectors and income-generating activities as they perceive it to be more efficient under the enduring climate change risks.

Regarding the gender role of decision-making, the number of adult males in the household is positively associated with the probability of IMV adoption in male and joint decision-making households. Meanwhile, the number of adult females in the household is negatively associated with the probability of IMV adoption in female and joint decision-making households. Again, this could be explained by the gendered division of labour in maize production, which is dominated by the ox-plow cultivation system led by male cultivators.

We found that the age of head is negatively associated with the probability of IMV adoption in joint decision-making households. This is consistent with the findings of Ndiritu et al. (2014) and Yirga et al. (2017). A possible explanation for this is that older household heads with longer farming experience are more likely to make unilateral decisions to adopt IMVs without seeking cooperation from their spouse. Education of household head is positively associated with the probability of IMV adoption in female decision-making households. This is consistent with the findings of Muriithi et al., 2018. This suggests that females living in more educated female-headed households are able to acquire and use the information on more maize varieties so that they can make the best decision given more diverse options.

Size of land holding and access to credit are positively associated with the probability of IMV adoption in male decision-making households. Distance from the market is negatively associated with the probability of IMV adoption in male and female decision-making households, while extension contact is positively associated with the probability of IMV adoption in male and joint decision-making households. Market information and participation in farmers training are positively associated with the probability of IMV adoption in male, female, and joint decision-making households. However, off-farm income is negatively associated with the probability of IMV adoption in joint decision-making household.

#### 4.2.2 Intensity of adoption

In this section, we present the results of the intensity of IMV adoption using a truncated regression model (Table 3). Of the variables included in the pool sample models, adult male, size of landholding, market information, and oxen owned positively and significantly determine the intensity of IMV adoption. Meanwhile, the interaction between female-headed and joint decision-making model, the age of the head, education, credit access, participation in social events, and distances from the main market negatively and significantly determine the intensity of IMV adoption. Both male- and female-headed joint decision-making households are older in age (see Table 1). A possible explanation for the negative interaction between female-headed and joint decision-making is that the female-headed joint decision-making households tend to refrain from using IMV seeds on their maize plot more than their male counterparts as part of their strategies to reduce risks related to the production of the drought-intolerant IMVs. Regarding age, older females are also more risk-averse than older males. Another possibility is that female-headed joint decision-making households may be economically less secure than their male counterparts. As a result, the former is less capable of investing in IMVs than the latter.

Regarding the gender role of decision-making, the number of adult males is positively related to the intensity of IMV adoption in all male, female, and joint decision-making households. This implies that an additional number of adult male labourers in the household increases the amount of maize area allocated to IMVs for male, female, and joint decision-making households. Age of head is negatively associated with the intensity of IMV adoption in joint decision-making households. According to Ndiritu et al. (2014), aging can be associated with shorter-term planning, loss of energy, and a tendency to be risk-averse. Hence, the negative association between the intensity of IMV adoption and joint decision-making in older headed households may be related to older farmers' higher preference of risk aversion associated with drought, disease and insect pests in maize production. The education level of the head is negatively associated with the intensity of IMV adoption in joint decision-making households. This could suggest that female and male family members living with more educated household heads are more aware of cultivating traditional maize varieties for risk mitigation purposes.

Land size is negatively associated with the intensity of IMV adoption in joint decision-making households. This suggests that any additional maize land is diverted to other local maize varieties. The positive relationship between oxen and intensity of adoption in all male, female, and joint decision-making households implies that an additional number of oxen increases the amount of land allocated to IMVs. This result confirms the finding from Asfaw et al. (2012). Credit is negatively associated with the intensity of IMV adoption in male and joint decision-making households. This result is consistent with our prior hypothesis suggesting that access to credit sources could change the interest of the farm household to invest in an off-farm business. Participation in social events is negatively associated with male and joint decision-making households. This could suggest that if farmers spend more time participating in different types of social events, they could have less time for maize farming operations. Participation in farmer training is positively associated with the intensity of IMV adoption for male decision-making households. This implies that participation in farmers training has a more educational effect, particularly for male decision-making households to adopt new agricultural technology like IMVs.

## 5 Conclusion and policy implications

Gender difference influences agricultural technology adoption in different ways, often determined by culturally defined gender roles and labour divisions. In some regions of Africa, men and women in the same household manage separate plots while in other regions, plots are managed jointly by male and female family members. Previous studies on agricultural technology adoption either do not consider gender dimensions of farming at all or simply conceptualize the household headship as a gender indicator. They fail to capture the actual gender power relationship over the relative positions of men and women in the same household in agricultural decision-making. Moreover, most of the studies that consider the individual plot manager as a gender indicator are based on the premise that men and women in the same household manage separate plots; they fail to examine the prevalent case of farm households where men and women manage the same plot of land jointly. To address these shortcomings of extant studies, our study examined a gender-based variation of decision-making around agricultural technology adoption using the data of male dominant, female dominant and joint decision-making households and IMV adoption in Dawuro zone of southern Ethiopia.

While we could not identify a gender difference in the rate of IMV adoption, we found a statistically significant gender difference in the intensity of IMV adoption. The intensity of IMV adoption is significantly higher with maize plots managed by male decision-making households and lower with plots managed by joint decision-making households. This result is in contrast to previous studies which found that joint decision-makers managing the same field adopted a new agricultural technology more intensively than individual decision-makers. Our results should be carefully interpreted in light of the following two conditions. First, the hybrid seed distributed to the farmers in all agro-ecologies of the study area is susceptible to drought, disease, and insect pests. Accordingly, farmers in the study area desired seeds which are resistant to adverse environmental conditions and associated pest attacks. Many of these farmers are using their own seeds, which they believe are adaptable to such hostile conditions. Second, the average landholding size of joint decision-making households is smaller than male and female decision-making households. In order to compensate for this economic disadvantage, joint decision-making households need to rely on more off-farm work than on increasing the intensity of IMV adoption, for fear of confronting the aforementioned ecological limitations of IMVs. Henceforth, policies and programs should be directed at developing and disseminating drought adaptive and disease resistant IMVs, most importantly for supporting economically less endowed but more gender egalitarian joint decision-making households. Farmer training and other extension programs should also promote integrated pest management programs aimed at shoring up the ecological fragility of the existing IMVs.

The first hurdle econometric results show that, after controlling other explanatory variables, neither the gender-differentiated headship nor the gender of decision makers in the household has a significant effect on the rate of IMV adoption. However, the second hurdle results show that the gender of the female headship and joint decision-making negatively and significantly influences the intensity of IMV adoption. This suggests that the intensity of IMV adoption in joint decision-making households headed by females is less than that those living in male-headed households. Thus, policy and program design need to support joint decision-making in female-headed households.

The number of adult males in the household is positively associated with IMV adoption while that of adult females is negatively associated. Age of household head has a negative effect on IMV adoption in joint decision-making households. Participation in social events also has a negative effect on the intensity of technology adoption for male and joint decision-making households. Most of the other explanatory variables included in the models show the expected results.

Taken together, our results on gender-based decision-making for IMV adoption and their implications for future research, policy and programs could be taken into consideration in order to help improve agricultural technology adoption as well as increase agricultural productivity for heterogeneous farm households in the developing world. We suggest that future studies on gender-differentiated agricultural technology adoption should be based on multiple technological packages that can simultaneously apply in the production process. This is in line with the recognition that the household adoption of one technology can influence their decision to adopt other technology.

## Footnotes

1 Joint decisions are understood to be made equally between male and female family members, in particular between husband and wife, in the same household.

2 Sharecropping is a form of farming in which a landowner allows another farmer (tenant) to use his/her land in return for a portion of crop produced. In Dawuro community, sharecropping is based on the customary rule. For example, the proportion of crop share and cropping term depends on their agreement based on the rules traditionally developed and abided by.

3 Kebele is the smallest administrative unit next to Woreda (district) in Ethiopia.

4 Only hybrid variety seeds were distributed to farmers in the study area. Thus, in this study, only fresh hybrid seeds were considered as ‘improved’ while recycled seeds were considered as ‘local’ varieties.

5 In this study, household head is defined as an individual who administers the household or individual who is considered to be responsible for the other household members in the household (Ragasa et al., 2012).

6 An agricultural development agent (DA) in Ethiopia is also known as an ‘extension agent’ working at the kebele level. Three DAs are assigned in each kebele to provide effective extension services closer to the farmers. Agricultural extension implementation modality differs slightly from region to region in Ethiopia. Regional states also differ in terms of how they supply agricultural inputs to farmers. In SNNP regional state, improved maize variety seeds are supplied to farmers through extension agents (Leta, Kelboro, Stellmacher, & Hornidge, 2017).

7 Different reports, including national statistics, show that holders with up to 10 ha of land are considered smallholders in Ethiopia. In this study, the maximum land holding size is 10 ha; hence all the sampled household are smallholder farmers.

8 In this study, family members are considered as individuals who are living in the same household. Our samples do not include maize farms managed jointly by members of separate households.

9 In Ethiopia, there are two crop production seasons: Meher and Belg. ‘Meher’ is the main crop season between Septembers and February, and ‘Belg’ is between March and August. Major cereal crops including maize are grown during the ‘Belg’ season and harvested during the ‘Maher’ season (CSA, 2017).

10 As noted in the extensive literature studied by Doss (2006), the definition for ‘adopter’ varies across studies. The appropriateness of each definition may depend on the specific context. A farmer may be defined as an adopter if she/he grows improved varieties as well as some local varieties. In our study area, farm households grow improved and local maize varieties either exclusively or in combination. Hence, in this study; we defined adopter as farm households growing improved maize varieties regardless of what else they grew.

11 In the study area, the IMVs are supplied to farmers only through government agricultural extension agents' outlet. They distribute different IMVs for different agro-ecologies.

12 In this study, area of landholding is equal to area of land owned by the households. Because all sampled households reported that their land is their own, not rented.

13 Tropical Livestock Units are livestock numbers converted to a common unit, Harvest Choice (2015). Conversion factors are cattle = 0.7, sheep = 0.1, goats = 0.1, pigs = 0.2, chicken = 0.01.

14 The IMVs seeds supplying are enclosed typically in a pack of 12.5 kg of seeds.
